# UV-Casting on Methacrylated PCL for the Production of a Peripheral Nerve Implant Containing an Array of Porous Aligned Microchannels

**DOI:** 10.3390/polym12040971

**Published:** 2020-04-22

**Authors:** Ruth Diez-Ahedo, Xabier Mendibil, Mari Carmen Márquez-Posadas, Iban Quintana, Francisco González, Francisco Javier Rodríguez, Leyla Zilic, Colin Sherborne, Adam Glen, Caroline S. Taylor, Frederik Claeyssens, John W. Haycock, Wandert Schaafsma, Eva González, Begoña Castro, Santos Merino

**Affiliations:** 1 Tekniker, C/Iñaki Goenaga 5, 20600 Eibar, Spain; ruth.diez@tekniker.es (R.D.-A.); xabier.mendibil@tekniker.es (X.M.); maricarmen.marquez@tekniker.es (M.C.M.-P.); iban.quintana@tekniker.es (I.Q.); 2 Laboratory of Molecular Neurology, Hospital Nacional de Parapléjicos, Finca. la Peraleda s/n, 45071 Toledo, Spain; francisco.gzlez@gmail.com (F.G.); fjrodriguez@sescam.jccm.es (F.J.R.); 3 Department of Materials Science & Engineering, University of Sheffield, Sheffield S3 7HQ, UK; leyla.zilic@gmail.com (L.Z.); c.sherborne@sheffield.ac.uk (C.S.); dradamglen@gmail.com (A.G.); c.s.taylor@sheffield.ac.uk (C.S.T.); f.claeyssens@sheffield.ac.uk (F.C.); j.w.haycock@sheffield.ac.uk (J.W.H.); 4 Histocell S.L., Parque Tecnológico de Bizkaia, 801 A, 2, 48160 Derio, Spain; wandertschaafsma@gmail.com (W.S.); egonzalez@histocell.com (E.G.); bcastro@histocell.com (B.C.)

**Keywords:** peripheral nerve, biopolymer, scaffold, microchannels, porosity, polycaprolactone, neuronal cells, Schwann cells

## Abstract

Peripheral nerves are basic communication structures guiding motor and sensory information from the central nervous system to receptor units. Severed peripheral nerve injuries represent a large clinical problem with relevant challenges to successful synthetic nerve repair scaffolds as substitutes to autologous nerve grafting. Numerous studies reported the use of hollow tubes made of synthetic polymers sutured between severed nerve stumps to promote nerve regeneration while providing protection for external factors, such as scar tissue formation and inflammation. Few approaches have described the potential use of a lumen structure comprised of microchannels or microfibers to provide axon growth avoiding misdirection and fostering proper healing. Here, we report the use of a 3D porous microchannel-based structure made of a photocurable methacrylated polycaprolactone, whose mechanical properties are comparable to native nerves. The neuro-regenerative properties of the polymer were assessed in vitro, prior to the implantation of the 3D porous structure, in a 6-mm rat sciatic nerve gap injury. The manufactured implants were biocompatible and able to be resorbed by the host’s body at a suitable rate, allowing the complete healing of the nerve. The innovative design of the highly porous structure with the axon guiding microchannels, along with the observation of myelinated axons and Schwann cells in the in vivo tests, led to a significant progress towards the standardized use of synthetic 3D multichannel-based structures in peripheral nerve surgery.

## 1. Introduction

Nearly 3% to 10% of people involved in a traumatic accident present traumatic peripheral nerve injury (PNI), which equates to almost one million patients per year in Europe and the U.S.A. needing surgical reconstruction of their nerves [1,2]. Peripheral nerve surgery has been proven to be a life changing surgery, improving the sensory function, mobility, and ability of patients to cope with normal life, affecting directly their physical and psychological well-being, and in the U.S.A. this surgery accounts for approximately $150 billion spent in annual health care [3,4].

The peripheral neurons show a good regeneration potential after injury [5], allowing the application of direct nerve repair and joining both ends of a sectioned peripheral nerve with epineural micro-sutures to treat PNI. However, this surgical procedure is limited to injuries showing distal to proximal nerve gaps smaller than eight millimeters [6]. For larger gaps, where a direct join of both nerve stumps is not possible, grafting is the alternative, with the autograft as the preferred choice [7,8]. The main drawbacks of this method are the possible mismatch between the diameters of injured and donor nerves, the additional surgical procedure, the low supply of nerves at the donor site and the consequent morbidity [9]. To overcome the problems the use of autograft implies, several studies have been carried out to develop an appropriate nerve guidance channel (NGC) able to heal PNI with long gaps [10]. 

It is well stablished that NGCs promote Schwann Cell (SchC) migration and axon growth, while guiding them between both ends of injured nerves. Hollow tubes of different materials used as NGCs are available on the market, e.g., NeuraGen, made of semipermeable collagen; Neurotube, which is a porous tube made of polyglycolic acid; Neurolac made of poly-DL-lactic-ε-caprolactone; and Neuroflex, which is a nano-porous tubular matrix made of collagen [11]. Other NGCs that are at the research stage and can be found in the literature are made from a wide variety of materials and their combinations, including chitosan, alginate hydrogels, agarose, poly-hydroxy alkanoates, poly-L-lactic acid, and poly-ε-caprolactone [12,13,14,15,16,17].

Currently, commercial NGCs do not offer the proper physical nor spatial guidance to the migrating SchCs and growing axons, eventually leading to a disorganized growth of axons, which limits their practical use to nerve gaps smaller than one centimeter [10]. To prevent the undesired effects that appear due to the lack of inner guidance in the lumen of the NGC, several studies suggested the use of an inner micro-channeled structure to support and promote the guided growth and protect the healing neural tissue [18,19].

The ideal NGC should be micro-channeled to stimulate the guided growth of axons, with channels of approximately 200 to 300 µm [20]. They should be porous with a suitable pore size to allow the interchange of nutrients for proliferating and growing cells and at the same time deter the axon cross-over and the infiltration of macrophages [21]. They should also have suitable mechanical properties to mimic the host tissue and an appropriate degradation rate to be resorbed at the same pace as the nerve heals.

Diverse and complex approaches can be found in the literature to manufacture the inner structure of an NGC. Huang et al. [22] used directional freeze drying to produce microchannels of collagen/chitosan assembled in an electrospun poly-ε-caprolactone (PCL) sheath. Lee et al. [23] used drawn sucrose fibers to subsequently electrospin PCL blends over them to finally dissolve the sucrose obtaining the microchannels. Pawelec et al. [20] used a dip coating method to individually form the microchannels and the outer tube, that were eventually assembled forming a bundle of microtubes inside the outer tube. Other examples implied the preparation of micro patterned films and posterior roll up to form a cylinder with a structured lumen [24]. All those methods involve complex laboratory techniques with low repeatability and low throughput that deter their scaling up and the effective implantation at the industrial level.

Here, we applied a simple UV light mold-casting method to manufacture highly porous micro-channeled scaffolds up to 5 cm length. Methacrylated PCL (PCLm) was injected and UV cured inside a flexible mold containing a configurable bundle of commercial stainless steel wires, easing the variation of the overall dimensions of the scaffolds and configuration of the microchannels. Two different approaches were applied to provide the scaffold with porosity, solid particle leaching, and high internal phase emulsion (HIPE) methods [25]. 

Porous micro-channeled scaffolds made of PCLm with tunable properties and porosities were obtained. These implants were successfully implanted in vivo in a 6 mm gap rat sciatic nerve model, which approximates a 1.6 cm gap in humans [5], showing the feasibility of this method to produce NGCs and its flexibility to tailor the properties of the scaffold through varying simple steps during the manufacturing process.

## 2. Materials and Methods

### 2.1. Materials: Synthesis and Characterization of Methacrylated Polycaprolactone 

The methacrylate functionalized oligomer used for the PCL was prepared using a commercially available PCL triol (*M*_n_ 900 g/mol, Sigma-Aldrich, Poole, UK). Within a 2 L round bottomed flask, PCL triol (1 molar equivalent, 30 g) was dissolved in 300 mL dichloromethane (DCM), (1:10 ratio of PCL:DCM). Triethylamine (TEA, 6 molar equivalent, 20.02 g, Sigma-Aldrich) was added and the solution was cooled in an ice bath for 30 min. Methacrylic anhydride, (MAA, 6 molar equivalent, 30.8 g, Sigma-Aldrich) dissolved in 50 mL DCM, was added dropwise using an addition funnel. The reaction solution was brought up to room temperature and left to react for 24 h. After, the solvent and unreacted TEA/MAA were removed under reduced pressure by rotary evaporation and the PCLm was purified by dissolving and precipitating in methanol four times at −80 °C. The final product was obtained by precipitating and removing the residual methanol. The PCL was aliquoted and stored at −20 °C. (1H) NMR spectroscopy analysis was performed on an AVANCE III (Bruker, Coventry, UK) spectrometer at 400 MHz to verify the methacrylation peaks of the PCLm. The spectra were analysed using MestReNova software, and all chemical shifts were referenced to the deuterated chloroform CDCl_3_ which was used as diluent (reference peak at 7.26).

### 2.2. Sample Design and Characterization

#### 2.2.1. Microfabrication of 3D Conduits

**Figure 1:**
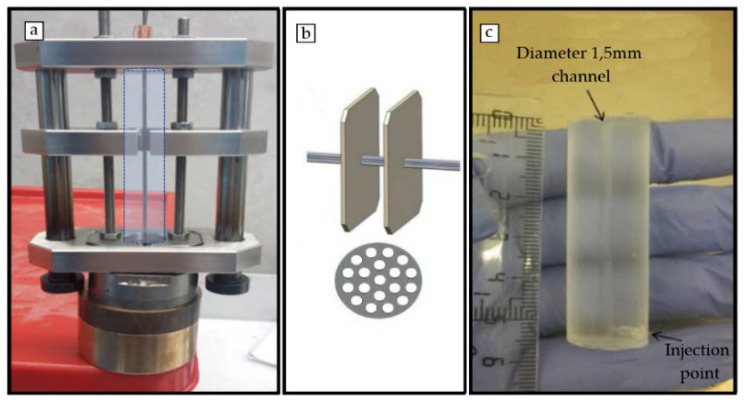
(**a**) Mechanical optimized setup for the 3D conduits fabrication in which the dashed line shows the position of the polydimethylsiloxane (PDMS) container. (**b**) Computer Aided Design (CAD) image showing distribution of the 200 µm wires arrangement through two opposite aligned micro perforated plates. (**c**) PDMS container.

A setup for the fabrication of 3D micro-channeled conduits made in PCLm was designed and fabricated in stainless steel (Figure 1a). It was optimized for optimal robustness and ease of assembly. The set up comprised a UV transparent polydimethylsiloxane (PDMS) container (Figure 1c), a set of stainless steel wires and an engineered system for robust alignment of the device and the wires. The set up consisted of two opposite aligned micro-perforated plates with micro-holes (cones of internal diameter of 230 µm and outer diameter of 210 µm to facilitate the insertion of the wires) crossed by 200 µm stainless steel wires aligned between the two plates (Figure 1b). The geometry obtained in the final longitudinal channels was defined by the number and diameter of the wires in the micro-perforated plates. This can be designed and manufacture as desired. The wires were crimped at each side with a copper ferrule to remain aligned. The straightness and stiffness of the wires were important to avoid the wires touching the PDMS container and/or each other. The PDMS container was aligned and surrounded the wires (Figure 1a). Its length and internal diameter, respectively, determined the length and diameter of the final manufactured device. The system was guided by a set of plates and guide lines. Upon assembly of the set up; the PCLm was blended with the photoinitiator 2,4,6-trimethylbenzoyl phosphine oxide/2-hydroxy-2-methylpropiophenone blend, Sigma-Aldrich), injected through the injection point of the PDMS container, and photo-crosslinked under UV-radiation at 350–450 nm. Finally, the setup was disassembled to obtain the 3D conduit. 

#### 2.2.2. Porosity Creation

Porosity was introduced following two different approaches: the use of solid leachable particles (glucose and sodium chloride) as a porogenerator and high internal phase emulsion from the methacrylated polycaprolactone.

*Glucose and sodium chloride porogens*: Reagent grade 99.5% purity anhydrous D-(+)-glucose and sodium chloride (NaCl) grade > 99.0% purity was provided by Sigma-Aldrich. Glucose or sodium chloride powder was sieved by means of stainless-steel wire strainers from CISA with a filter size between 20 and 50 µm and mechanically shaken. Sieved glucose/sodium chloride was stored in desiccator at room temperature and was mixed with PCLm and 2 wt% photoinitiator (2,4,6-trimethylbenzoyl phosphine oxide/2-hydroxy-2-methylpropiophenone blend, Sigma-Aldrich) in a certain proportion in weight (50:50; 60:40; 70:30 glucose or sodium chloride: PCLm). The mixture was injected into the PDMS container.

*High Internal Phase Emulsion (HIPE)*: The PCLm was prepared using an adapted protocol as described recently [25,26]. In brief, 0.6 g of the methacrylated PCL was dissolved in 0.4 g of a 60:40 blend of chloroform and toluene. Surfactant was added to the solution (Hypermer B246, 10 wt% relative to monomer, i.e., 0.06 g). Photoinitiator (2,4,6-trimethylbenzoyl phosphine oxide/2-hydroxy-2-methylpropiophenone blend) was added as 5 wt% relative to the PCL oligomer (0.03 g). The solution was heated to 35 °C while water was added dropwise during constant mixing (6 mL water, 350 rpm). The produced HIPE had a white foam consistency which could then be injected into the PDMS container. 

#### 2.2.3. Micro Computed Tomography (MicroCT) of Nerve Guide Conduit

MicroCT was performed on a Skyscan 1272 (Bruker). The NGC samples were secured vertically by coating the brass platform surface with dental wax and inserting 1 mm of the NGC into dental wax in order to stabilize the sample against toppling during rotation. The samples were scanned without any filters with a voxel size of 9 µm^3^ at 50 kV/200 µA, in 0.7° increments, with an exposure time of 303 ms over 360° of the sample. The scanned images were cropped to select only the samples, reconstructed, analysed, rendered, and visualised with Nrecon (v1.6.9.8 Bruker, using the Feldkamp algorithm), a CT analyzer (v1.14.4.1, Bruker), CTvol (v2.2.3.0 Bruker), and CTvox (v2.7.0 r990, Bruker), respectively. In order to minimise the well-known CT reconstruction artefacts, all samples were subject to misalignment compensation and beam hardening correction which prevents the mis-calibration of cells in the digital x-ray detectors and image streaking caused by adjacent high attenuation objects.

#### 2.2.4. Mechanical Characterization of 3D Conduits

3D conduit samples were immersed in phosphatebuffer saline (PBS) solution for 10 min and clamped into a tensiometer (BOSE Electroforce test instruments, TA Instruments, Eden Prairy, MI, USA, using a 450 N load cell). A single pull to failure test was carried out at a speed of 0.1 mm/s. The first failure point or plateau was used to calculate the ultimate tensile strength (UTS), and the initial linear gradient was used to calculate the Young’s modulus. For all specimens, the Young’s modulus was determined from the initial length and area of the specimens.

#### 2.2.5. Differential Scanning Calorimetry (DSC). Ageing Study of 3D Conduits

Thermal analysis of polymers and blend was conducted using DSC 1/500 (Mettler Toledo, Leicester, UK) equipped with Intra-cooler IC70 cooling system. We heated 5 mg of polymer from −70 to 200 °C, at a heating rate of 10 °C per min. Leached and unleached samples were measured at time zero which corresponded with 2 weeks from the processing, and at time 1, aged for 4 months in a desiccator at room temperature.

#### 2.2.6. Biodegradation Analysis

Following the ISO 10993-13, the samples were manufactured and sterilized using gamma irradiation (25 kGy). Before start of the degradation assay, the initial weights of the samples were determined using a precision balance after the samples were dried overnight at 37 °C in a vacuum. The changes in weight were applied for the determination of the degree of degradation.

The samples were fully immersed in closed Falcon tubes in 3% hydrogen peroxide solution (H_2_O_2_) (described in ISO10993.13-4.1.4.1.3a). To maintain radical concentration, 3% H_2_O_2_ (0.88M) solution was changed weekly for the entire duration of the experiment and no metal ions were added to catalyse the H_2_O_2_ degradation [27]. The final sample weight was measured after 1, 3, and 6 months with a minimum of *n* = 4 samples per material, and, in parallel, a hydrolytic degradation was performed using laboratory grade water with *n* = 1. Before measuring the final weight, the samples were again dried overnight at 37 °C in a vacuum.

### 2.3. In-Vitro Experiments and Analysis

#### 2.3.1. Culture of Cell Lines

L929 murine fibroblast cells were cultured in high glucose Dulbecco’s modified Eagle’s medium (DMEM; Sigma-Aldrich) supplemented with 10% fetal bovine serum (FBS; Biochrom, Cambridge, UK) and 1% Penicillin-Streptomycin (Pen/Strep; Lonza, Basel, Switzerland). Human neuroblastoma cells SH-SY5Y were cultured in Advanced DMEM/F12 (Gibco, Dublin, Ireland) supplemented with 10% FBS and 1% Pen/Strep. NG108-15 neuronal cells, (obtained from ECACC, Porton, UK) were cultured in Dulbecco’s modified Eagle’s medium (DMEM) containing 10% fetal calf serum, 1% penicillin/streptomycin, 1% glutamine, and 0.5% amphotericin B. Cells were used for experiments between passages 11–15. All cell cultures took place in a humidified atmosphere at 37 °C, 5% CO_2_. For the maintenance of the cells, they were passaged when a confluency of 80%−85% was reached. Passaging was done by 5 min of trypsinization (0.05%; Invitrogen, Waltham, MA, USA) at 37 °C. Thereafter, the cells were processed as below.

#### 2.3.2. Cytotoxicity Analysis of 3D Conduits

Cytotoxicity assays were performed according to the indirect 3-(4,5-Dimethylthiazol-2-yl)-2,5-diphenyl tetrazolium bromide (MTT) assay (Roche, Basel, Switzerland, Cell proliferation kit I) according to ISO 10993-5 guidelines. For this assay, a standard cell culture medium was conditioned for 24 h with the materials of interest or negative control (high-density polyethylene, United States Pharmacopeia (USP)) and positive control (positive bioreaction, USP). Considering the dimensions of the implants, the corresponding amount of cell culture medium according to ISO 10993-12 was added. All samples were sterilized by gamma irradiation at 25 kGy before the medium conditioning took place.

For all experiments performed under ISO 10993-5 conditions, the mouse fibroblast cell line L929 was used. In addition, when indicated, the experiments were performed with neuroblastoma cell line SH-SY5Y. All cell cultures and incubation steps were performed in a humidified atmosphere at 37 °C, 5% CO_2_ conditions. For MTT analysis, cells were seeded at a density of 10,000 cells/well in a 96 well plate. MTT was performed according to the manufacturer’s instructions. In short, after incubation with conditioned media or controls, the cells were incubated for 4 h with MTT, then the cell proliferation kit I solubilization solution was added for 24 h. The absorbance was measured at a wavelength of 550 nm using a Multiskan Ascent (Thermo Scientific, Göteborg, Sweden) plate reader. The cell viability was determined by the following formula: Viability = 100 × DO550 nm sample/ DO550 nm control. When the cell viability was reduced to <70% when compared to the negative control, the tested material was considered cytotoxic. 

#### 2.3.3. In Vitro Analysis of PCLm Polymer Films

*NG108-15 neuronal cell differentiation and cell viability on PCLm films*: Up to 40,000 NG108-15 neuronal cells were cultured on PCLm flat films for 6 days. The medium was changed every 2 days and switched to serum free DMEM on day 2 to promote neurite outgrowth from cells. NG108-15 neuronal cell differentiation was assessed as per the methods of Daud et al. [28]. Briefly, cells were washed with PBS, fixed with 3.7% (v/v) paraformaldehyde and permeabilized with 0.1% Triton X-100. NG108-15 neuronal cells were incubated with a mouse anti-*β* III-tubulin (neurite marker) antibody (1:250) (Promega, Southampton, UK) diluted in 1% BSA in PBS and incubated at 4 °C for 48 h. After washing with PBS, NG108-15 neuronal cells were labelled with Texas Red-conjugated anti-mouse IgG antibody (1:200 dilution in 1% BSA from Vector Labs, Peterborough, UK) and 4,6-diamidino-2-phenylindole dihydrochloride (DAPI) (Sigma-Aldrich) (300 nM) for 90 min at room temperature. The samples were imaged with an upright Zeiss LSM 510 confocal microscope, using a helium-neon laser (543 nm) for Texas Red excitation (λ_ex_ = 589 nm / λ_em_ = 615 nm) and a Ti:sapphire laser (800 nm) was used to image DAPI (λ_ex_= 358 nm / λ_em_ = 461 nm). The images were quantified to determine the percentage of neurite bearing cells, the average number of neurites per neuron and the average neurite length per condition. 

To assess the neuronal cell viability, the samples were stained with 0.001% Syto-9 (Invitrogen) and 0.0015% propidium iodide (Invitrogen) at 37 °C for 30 min. The samples were imaged using an upright Zeiss LSM 510 (Oberkochen, Germany) confocal microscope. Three fields of view were taken for each sample, and the cells were counted using ITCN cell counter software on NIH Image J (threshold = 40 to 90). The average number of live cells versus dead cells ± SD, per sample, was plotted on a graph, as well as expressed as a percentage of cell viability [28].

*Rat primary Schwann cell phenotype and viability*: Rat primary Schwann cells were isolated and cultured via methods from Kaewkhaw et al. [29] in which the sciatic nerves were dissected and dissociated. Using a specialized DMEM medium, Schwann cells could be expanded to higher passage numbers, whilst the medium inhibited fibroblast growth (via D-valine). Schwann cells between passages 4–7 were used for these experiments. To confirm the maintained phenotype, Schwann cells were labelled for S100*β* as per the methods of Daud et al. (2012) [28]. Briefly, the samples were washed, and the cells were fixed (as per above). The Schwann cells were labelled with a polyclonal rabbit anti-S100*β* (1:250) (Dako) diluted in 1% BSA in PBS and incubated at 4 °C for 48 h. After washing with PBS, the Schwann cells incubated with an FITC-conjugated secondary anti-rabbit IgG antibody (1:100 dilution in 1% BSA for S100 and p75 staining) 4,6-diamidino-2-phenylindole dihydrochloride (DAPI) (Sigma-Aldrich) (300 nM) for 90 min, at room temperature. The samples were imaged with an upright Zeiss LSM 510 confocal microscope, using an argon ion laser (488 nm) for FITC excitation (λ_ex_= 495 nm / λ_em_ = 521 nm) and a Ti:sapphire laser (800 nm) was used to image DAPI (λ_ex_ = 358 nm / λ_em_ = 461 nm). Images were quantified to determine the average Schwann cell length (phenotype marker) and the aspect ratio (length divided by width) [30]. 

Live/dead analysis was used to confirm the cell viability of primary Schwann cells cultured on the spin coated PCLm films while the average primary Schwann cell length was also calculated. We cultured 60,000 rat primary Schwann cells on the spin coated PCLm films for 6 days. The samples were stained with 0.001% Syto-9 (Invitrogen) and 0.0015% propidium iodide (Invitrogen) at 37 °C for 30 min, imaged, and quantified as per the method of [27].

### 2.4. In Vivo Experiments and Analysis

#### 2.4.1. In Vivo Nerve Model for Anatomical and Histological Analysis

Haematoxylin and eosin (H&E; Raymond A Lamb Ltd., Altringham, UK) staining of native and acellular nerve segments was used to evaluate tissue histoarchitecture. Six rat sciatic nerve samples, left and right, from three animals were immersed in Harris’s haematoxylin (Thermo Fisher Scientific Ltd., Altringham, UK) (1 min) and rinsed for 3 min under tap water for blueing. The slides were then immersed into eosin Y (VWR International, Lutterworth, UK) for 3 min, dehydrated, cleared, and mounted using Distyrene Plasticier Xylene (DPX) mountant before being viewed under Kohler illumination for transverse and longitudinal analysis. The average number of fascicles per nerve and average diameter, the number of average myelinated axons and averaged diameter per nerve were determined. 

#### 2.4.2. In Vivo Analysis of the 3D Conduit in a 6 mm Gap Rat Sciatic Model

*Animals*: Female Wistar rats (200–220 g) were used. The animals were housed in plastic cages, maintained at 22 °C in a 12 h light/dark cycle, and allowed to free access to water and food. The experimental procedures were approved by the respective Ethical Committee and followed the rules of the European Communities Council Directive. The Animal Research and well-being committee of National Hospital for Paraplegics in Toledo approved unanimously at the board meeting that took place on June 13, 2013 the research animal model, assigning it the reference number 106/2013.

**Figure 2:**
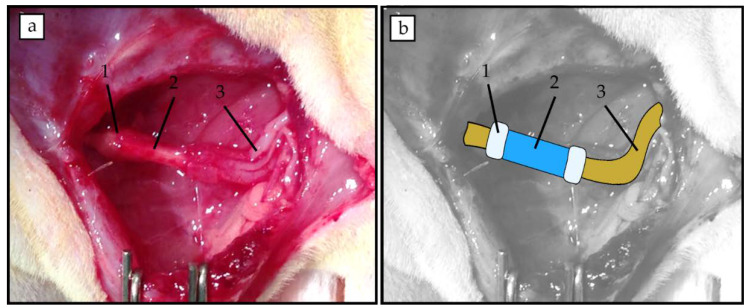
(**a**) Representative image of implanted PCLm 6 mm long tube held with fibrin for a 6 mm gap. (**b**) Superimposed schematic drawing highlighting the position of the fibrin joints (1), the micro channeled conduit (2) and the sciatic nerve stumps (3).

*Surgical procedure*: All surgical procedures were performed with aseptic operating conditions and under pentobarbital/xylacine anesthesia (40/10 mg/kg i.p. respectively). The right sciatic nerve (*n* = 4) was exposed and cut 6 mm distal to the exit of the gluteal nerve. We implanted 6 mm long devices by means of a silicone plug filled with fibrin/thrombin (Figure 2). The muscle plane was sutured with resorbable 5-0 sutures, and the skin with 2-0 silk sutures. The wound was disinfected, and the animals were treated with amitriptyline to prevent autotomy. The animals were followed-up for 4 weeks. After this time, the right sciatic nerves were exposed and cut 6 mm distal to the distal end of the silicone tube. Then, the proximal cut stump was dipped in 2 μL of the tracers (True Blue at the tibial branch and 1,1’-Dioctadecyl-3,3,3’,3’-Tetramethylindocarbocyanine Perchlorate (DiI) at the peroneal branch) placed in a Vaseline pool for 1 h. One week later, the animals were perfused and dorsal root ganglia (DRG) (L4, L5), lumbar spinal cord section, and the regenerated sciatic nerves were obtained for analysis.

*Histological Analysis:* After perfusion, the regenerated nerves were collected and post-fixed in 3% glutaraldehyde. The nerves were post-fixed in osmium tetroxide (2%) and glucose (10% in PBS 0.2M) (1:1), 90 min at room temperature. The nerves were washed three times with distilled water and kept overnight at room temperature. The nerves were dehydrated in ascending series of acetone and embedded in Araldite resin. The nerves were sectioned using a ultramicrotome in 1 μm sections. The collected sections were stained with toluidine blue and examined under light microscopy. Images of the whole nerve were acquired at 4× with a digital camera, while sets of images were chosen randomly to represent at least the 30% of nerve cross-sectional area were acquired at 60× from the mid and distal parts of the regenerated nerves. Measurements of the cross-sectional area of the whole nerve and the estimation of myelinated fibers were performed by using Image J Software.

*Immunohistochemistry (IHC):* Regenerated nerves were processed for IHC analysis. Cross sectional sections (30 μm) of the regenerated nerves were used to label regenerated myelinated axons (neurofilament 200), Schwann cells (S100*β*) and cells nuclei (DAPI).

### 2.5. Statistical Analysis

GraphPad was used to perform the statistical analysis on the collected data. One-way analysis of variance with a Bonferroni post hoc test was conducted to analyze differences on histological analysis. The data were considered significantly different when *p < 0.05*.

## 3. Results and Discussion

### 3.1. Physical Dimension of Rat Sciatic Nerve

**Figure 3:**
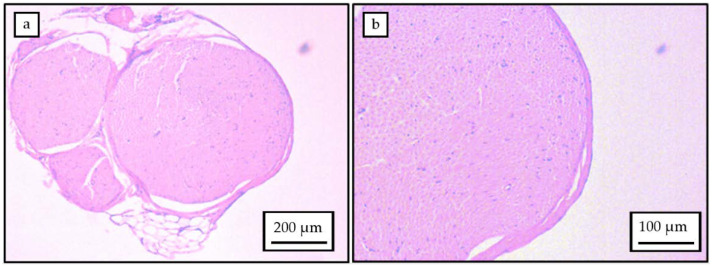
(**a**) Transverse section of rat sciatic nerve showing the four fascicles. (**b**) Myelinated axons on the transverse section of the rat sciatic nerve.

H&E analysis was performed on transverse sections of rat sciatic nerves. As the sciatic nerves are not perfectly circular, the epineural diameter was calculated as a range from the transverse sections, approaching the epineural diameter to a minimum of (900 ± 3 µm) and a maximum of (1183 ± 85 µm). The sciatic nerve was observed consistently to comprise of four fascicles (Figure 3), determined as the tibial, peroneal, sural, and cutaneous branches. Physical dimension measurements were performed for each of these categories and diameters established for each fascicle: tibial branch (692 ± 30 µm), peroneal branch (404 ± 44 µm), sural branch (252 ± 41 µm) and cutaneous branch (90 ± 9 µm). Considering the presence of all four nerve branches, the number of average myelinated axons approximated 6000, leading to an average diameter per nerve in the range of 1–10 µm. These figures are quite consistent with those previously reported [31]. The data therefore enabled an implant diameter of 1.5 mm to be defined, with microchannel widths in the range of several tens of micrometers for applications in rat sciatic nerve injuries. 

### 3.2. Material and Manufactured 3D Porous Implant Characterization

H-NMR analysis comparing the PCL to the PCLm showed a decrease in the PCL methylene groups around 3.6 ppm as they were converted to methacrylate groups in the PCLm corresponding to peaks around 6, 5.5, and 1.9 ppm (Figure S1). These findings relate to a previous published work on photocurable PCL [26].

**Figure 4:**
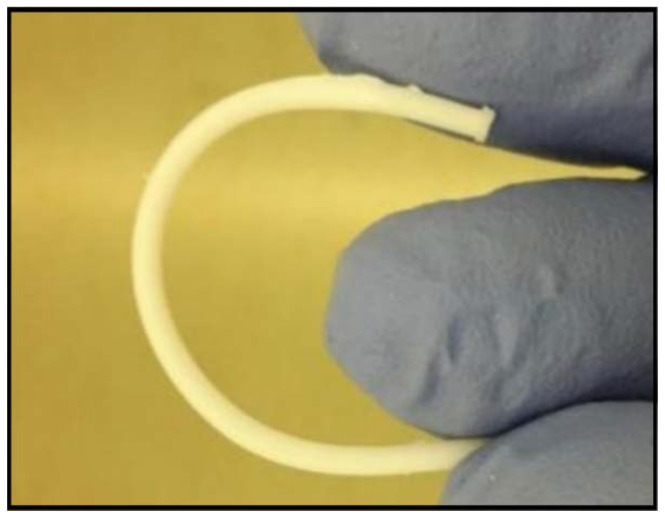
Picture showing the elastic behavior of a 5 cm long tube after leaching.

3D porous conduits were fabricated up to 50 mm long and 1.5 mm width, using sodium chloride or glucose as porogen, followed by leaching under deionized water. The channels were 200 µm width, the minimum value which was compatible with a robustness in fabrication and up to 5 cm long open channels for the whole conduit. The channels were fabricated through the insertion of 19 stainless-steel wires at the mechanical setup described in the Figure 1. Thus, 200 µm width microchannels were manufactured, maximizing the open volume within the array while the microchannels wall thickness is minimized [20] but they should still maintain their function as a structural support; detailed images of obtained microchannels quality and arrangement are shown in the Supplementary Materials (Figure S2). The 3D conduits shown were moderately elastic (Figure 4) with Young’s modulus fitted to different figures attending to the weight proportion of the porogen. 3D porous conduits with the same dimensions, and in which the porosity was created through the HIPE protocol were also manufactured. These were flexible, compressible, and showed a spongy behavior.

As the degree of porosity and porous size was selected, parallel considerations were made [10]. The conduit should protect the site of injury from the infiltration of the surrounding cells, while at the same time should retain a certain degree of porosity allowing the diffusion of soluble factors, oxygen, and nutrients through the tube wall, as well as affecting the migration and organization of myofibroblasts [32], which are responsible for the undesired synthesis of scar tissue. Thus, the capacity to limit and/or prevent the formation of the contractile capsule of myofibroblasts, as well as a certain grade of permeability are two key factors which must be carefully considered when working for the improvement of the tube wall properties. On the other hand, these requirements should also facilitate the need to prevent the infiltration of inflammatory cells into the conduit and to minimize the diffusion of growth factors out of the conduit. Accordingly, pore sizes in the range of about 20–30 µm were considered optimal [20,33], while pore sizes lower than 38 µm were shown to encourage glucose diffusion, and slow protein diffusion and cell migration [34]. In this work, the porogen size was fixed to 20–50 µm to ensure that the pores were interconnected as combined with a high degree of porosity, which as combined with the referenced polymer to porogen weight proportions and considering the successful leaching of the porogen, led to porous volume fractions ranging from 0.43 to 0.63 for the glucose porogen and 0.35 to 0.55 for the sodium chloride.

**Figure 5:**
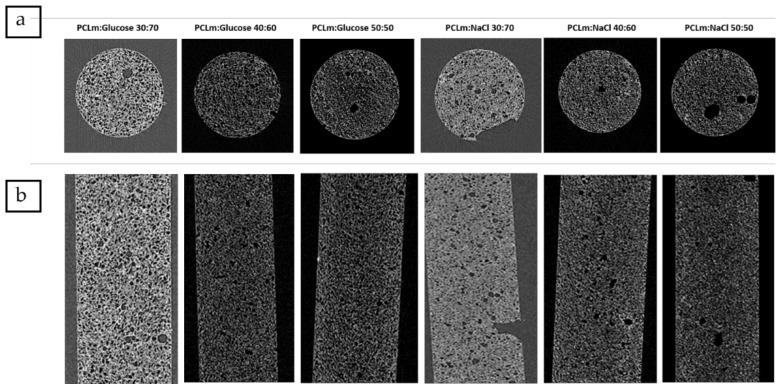
Micro computed tomography (MicroCT) analysis following different orientations: (**a**) transversal section and (**b**) longitudinal section of leached methacrylated poly-ε-caprolactone (PCL) (PCLm) UV cast cylinders mixed with different percentages of porogens (sodium chloride or glucose). The tubular structures shown are all 1.5 mm diameter and 5 mm in length.

**Figure 6:**
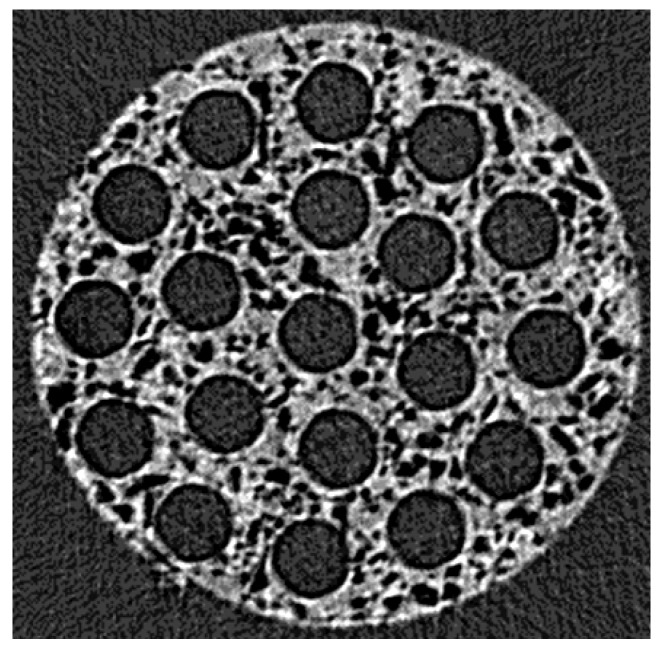
MicroCT image showing the transversal section at the middle of a 5 mm length implant containing microchannels of 200 µm.

A microCT analysis was developed for different concentrations of both porogens (Figure 5). The analysis was developed slide by slide in the transversal (Figure 5a) and longitudinal (Figure 5b) sections of single UV cast cylinders as manufactured without the presence of microchannels in their lumen. This showed that both sodium chloride and glucose were successfully leached at all concentrations, and porosity was found throughout the internal structure. By inference, this indicates that the pores must be interconnected as water would have no way to penetrate to the core of the material without such interconnectivity. The distribution of the porosity was maintained in implants containing microchannels as the transversal section of the microCT image (Figure 6) at the middle of a 5 mm implant contained microchannels. In order to ensure the efficiency of porogen removal, thermogravimetry analysis (TGA) indicated that, after the leaching process, less than 1% of porogen remains inside the manufactured conduit (data not shown). 

**Figure 7:**
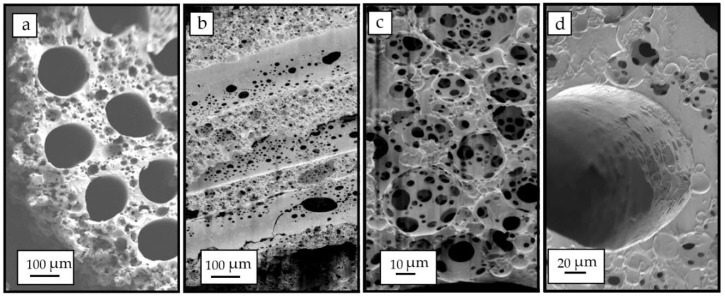
SEM images showing the transversal sections of the conduit showing the high-volume porosity created by high internal phase emulsion (HIPE) (**a**). Lumen of the microchannels after being cut and inspected by SEM, in which a high porous structure is observed (**b**), Detail of the porous structure of the manufactured implant (**c**), and detailed image of a single microchannel (**d**).

As the conduit was made porous through the HIPE templating, the porous volume increased estimating that this fraction goes well above 70% according to inspection of the Scanning Electron Microscopy (SEM) images of the transversal and longitudinal sections of the tube (Figure 7a,b). This was expected as, in the original emulsion, the water droplet phase was 83.3% of the polymer/solvent phase. The sponge-like conduits were easily cut for inspection and showed a porous size distribution in the lumen of the channels (Figure 7b). Porous sizes from 5 to 70 µm were observed but the largest proportion was found with 20–50 µm sizes. A pore size distribution like this was expected as the water droplets are broken into smaller ones at a rate depending on the mixer type and speed, and these affect the distance of the droplets from the stirrer as well as the stirring efficiency to break apart the larger droplets into smaller ones. Our scaled system uses a magnetic stirrer set at 350 rpm. Nevertheless, this pores size distribution gives a high packing efficacy of water droplets and consequently porosity within the sample. Higher magnification of the pores outside and inside the channels are shown in Figure 7c,d, respectively.

**Figure 8:**
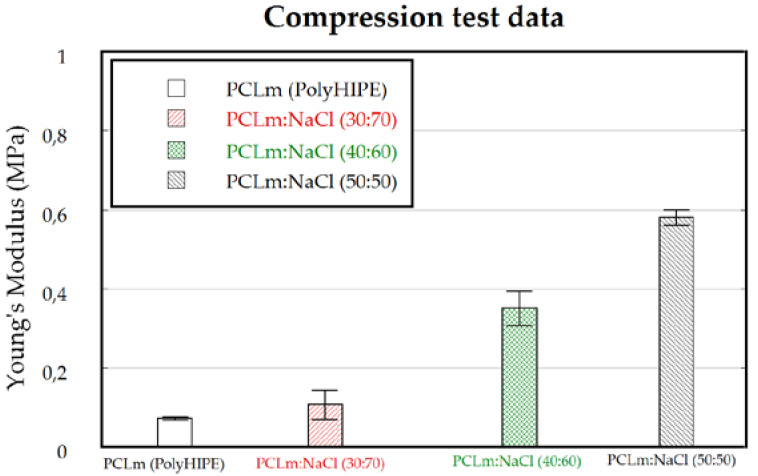
Compression testing of casted channeled nerve guidance conduits (*n* = 4). The Young’s modulus can be fitted to different nerve targets depending on the way the porosity is created.

The elasticity of the tube was shown to be modulated attending to the degree of porosity introduced in the 3D conduit. The Young’s modulus of the tube was measured for all configurations, showing that a wide range of flexibility (0.07–0.6 MPa) could be selected for mimicking different peripheral target nerves (Figure 8). These results are similar to previous works addressing the mechanical properties of the HIPE scaffold, in which an increased porosity decreased the Young’s modulus of the scaffold. An increased porosity means a decrease in the cross-sectional area, meaning less force is required for the same stress applied [25]. For the in vivo model, a 50:50 PCLm:NaCl porogen proportion with a Young’s modulus of (0.58 ± 0.02 MPa) was chosen being the most similar value to the previously reported Young’s modulus (0.58 ± 0.015 MPa) for a rat sciatic nerve [35].

**Figure 9:**
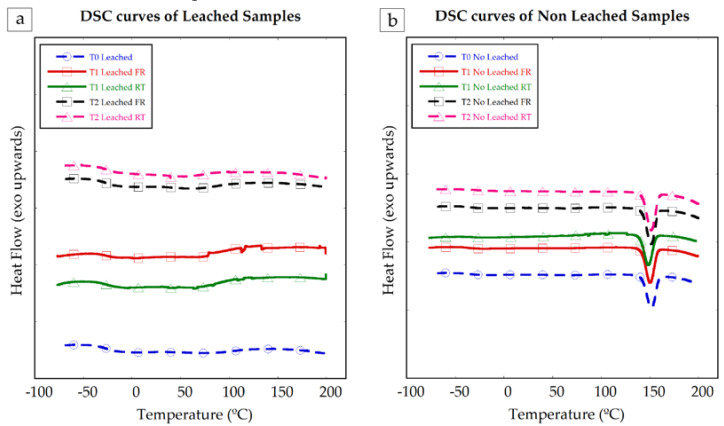
DSC curves of leached (**a**) and non-leached (**b**) samples of PCLm at different periods. DSC curve of leached and non-leached PCLm as processed in dashed line with circles. Samples of PCLm leached and non-leached aged for 1 month, stored in freezer (FR) continuous line with squares and stored at room temperature (RT) continuous line with triangles. Samples of PCLm leached and non-leached aged for 3 months, stored in freezer (FR) in dashed line with squares and stored at room temperature (RT) dashed line with triangles. Exothermic heat flow upwards.

Samples of the manufactured PCLm conduits were tested with differential calorimetry scanning (DSC) to check the stability of the samples along time, the appearance of crystalline structures, and the influence of the storage conditions. In Figure 9, the DSC curves of the leached (**a**) and non-leached (**b**) samples are shown. The samples were measured at T0, just after the casting and UV curing process, at T1, one month stored in a freezer (−20 °C) and in a desiccator at room temperature and at T2, three months stored in the freezer (−20 °C) and in the desiccator at room temperature.

Non-leached samples show clear endothermic peaks around 140 °C, consistent with the melting temperature of glucose, which was used as porogen in those samples. No clear peaks or curve slope changes attributable to the presence of crystalline structures or further polymer curing were observed. The PCLm samples were observed to be stable and did not show crystallization nor reticulation evolution within the tested time range.

### 3.3. Cytotoxicity and Biodegradability of 3D Conduits

**Figure 10:**
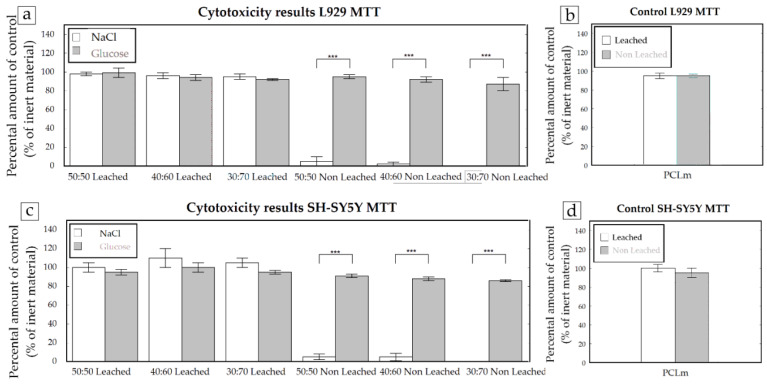
The cytotoxicity results using NaCl or glucose as porogens on PCLm conduits (*n* = 5). Cytotoxicity assays were performed with L929 (**a**) and SH-SY5Y (**c**) cells. As a control, PCLm cylinders were included without porogen treatment (*n* = 1) but, in every other way, treated the same (**b** and **d**).

The materials were tested for their cytotoxicity by an indirect MTT assay according to ISO 10993-5 guidelines. Thus, for each condition, five conduits were introduced into standard cell culture medium for 24 h and this extract was used afterwards for determining their cytotoxicity. Glucose leached and non-leached samples were compared to sodium chloride leached and non-leached samples. As a control, PCLm samples were included without the use of porogens but, in every other way, treated the same. The experiments were performed with the mouse fibroblast cell line L929 and human neuronal SH-SY5Y cells, and the cytotoxicity was assessed as previously described. Both the L929 and SH-SY5Y cells, after leaching both sodium chloride and glucose, were shown to be non-cytotoxic (Figure 10). However, when the samples were not leached, the use of NaCl showed severe cytotoxicity with >90% cell death. This effect was not observed in non-leached PCLm:Glucose samples. 

Taking in mind that due to processing, the possibility exists that some residual porogen may be present in the guidance tube, these results indicate that, from a cytotoxicity point of view, glucose would be the preferred porogen. However, as the 3D conduits were kept in deionizer water for 1 week with a daily change of solution, the sodium chloride porogen-based conduits do not show any cytotoxicity (Figure 10). Thus, as discussed previously, the 50:50 PCLm:NaCl porogen proportion was selected for in vivo validation. 

The 3D conduits in which the porosity was introduced through the HIPE protocol showed a lack of cytotoxicity (data not showed) when the samples were washed for 5 days in a 70% ethanol solution and rinsed for 24 h in a deionized water solution to remove any trace of unreacted photoinitiator.

**Figure 11:**
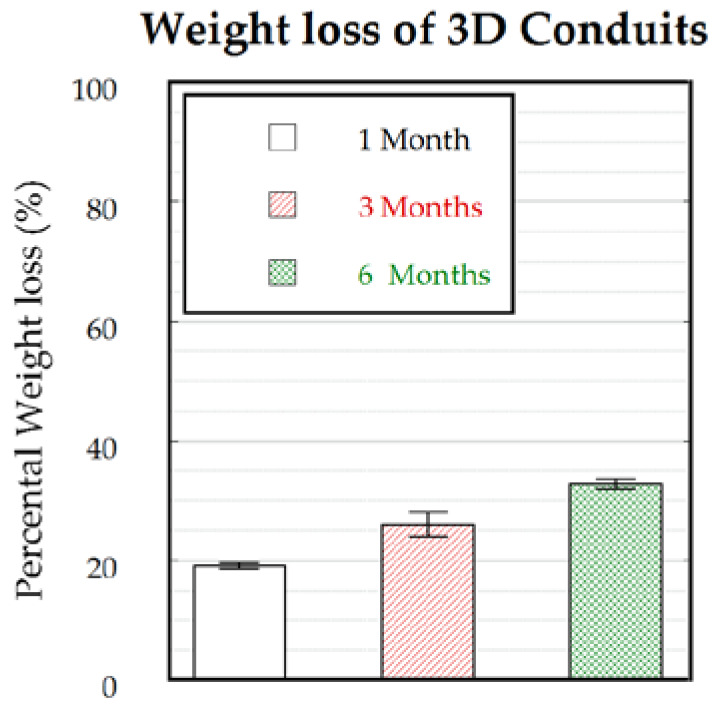
Weight loss of 3D conduits (*n* = 5) as immersed in a 3% H_2_O_2_ solution for 1, 3, and 6 months.

As the environment where the nerve guidance conduits will be implanted has an oxidative nature, the samples were analyzed by oxidative degradation. For this, five samples were immersed in a 3% hydrogen peroxide solution for the designated time points with a weekly change of solution. As a control, in parallel, a hydrolytic degradation was performed (*n* = 1). The 3D conduits showed a substantial weight loss (Figure 11) after 1 month (~20%). This substantial weight loss was due to the oxidative nature of the solution as PCLm subjected to hydrolytic degradation showed a <1% weight loss after 1 month of degradation (data not shown). Although the PCLm conduits showed relatively rapid degradation in 1 month, only an additional 5% weight loss was measured after 3 months, while less than 35% weight loss was observed in 6 months (Figure 11). Thus, considering that, in peripheral nerve injuries, the axon regeneration rate can approach figures around 1 mm/day in humans and 3 mm/day in rats [36], the biodegradation rate can be considered to provide protection to the regenerated axons from intervening scar tissue and restricting axon misdirection toward the distal nerve segment. A faster biodegradation rate may be desired to prevent a possible pain or inflammation due to the eventual collapse of the conduit. 

### 3.4. In Vitro Analysis of PCLm Films

**Figure 12:**
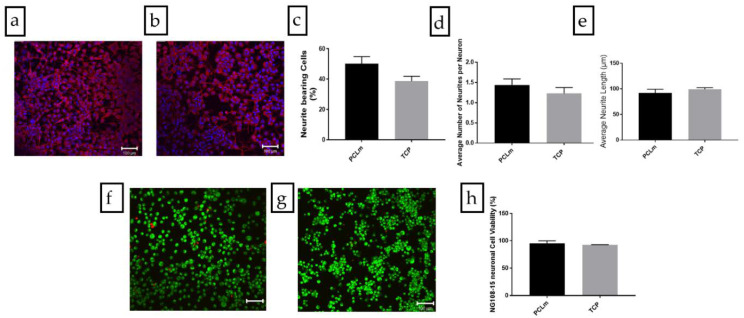
Confocal micrographs of the NG108-15 neuronal cells cultured on (**a**) PCLm films and (**b**) tissue culture poly-styrene (TCP) control labelled for β III tubulin (red) and 4,6-diamidino-2-phenylindole dihydrochloride (DAPI) (blue) (Scale bar = 100 µm). The images were quantified to determine (**c**) the percentage of neurite bearing cells, (**d**) the average number of neurites per neuron, and (**e**) the average neurite length per condition (mean ± SD, *n* = 3 independent experiments). Confocal micrographs of NG108-15 neuronal cells cultured on (**f**) PCLm film and (**g**) TCP controls labelled for Syto-9 (green) and propidium iodide (red) for live/dead analysis (Scale bar = 100 µm). The images were quantified to determine the cell viability as a percentage of live versus dead cells (**h**) (mean ± SD, *n* = 3 independent experiments).

NG108-15 neuronal cells were cultured on spin coated PCLm films and the cells were labelled for DAPI and βIII tubulin. Confocal micrographs were taken, and the images were quantified. Figure 12a shows the spread of NG108-15 neuronal cells on the film, in which long neurites can be appreciated as well as a high number of cells bearing neurites. We performed an analysis of the confocal micrographs to calculate the percentage of neurite bearing NG108-15 neuronal cells, (50 ± 5%) (Figure 12c) as well as the average number of neurites expressed per NG108-15 neuronal cell, which was estimated as 1.44 ± 0.15 (Figure 12d). The average neurite length was estimated with 100 neurites per condition around 92 ± 7 µm (Figure 12e). The NG108-15 neuronal cell viability was analyzed on PCLm spin coated films. Confocal micrographs of PCLm films were taken to analyze the biocompatibility (mean ± SD, *n* = 3 independent experiments), showing (Figure 12f–h) that the cell viability was well above 90% (95 ± 5%). These results led to the conclusion that the PCLm material supported the growth and maturation of the NG108-15 neuronal cells.

**Figure 13:**
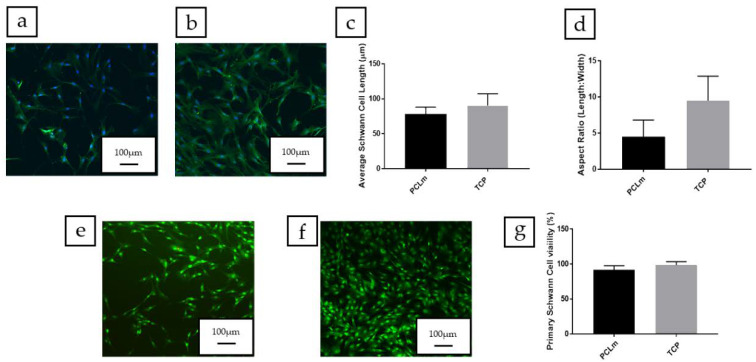
Confocal micrographs of rat primary Schwann cells cultured on (**a**) PCLm films and (**b**) TCP control labelled for S100β (green) and DAPI (blue) (Scale bar = 100 µm). The images were quantified to determine (**c**) the average Schwann cell length and (**d**) the aspect ratio of Schwann cells per condition (mean ± SD, *n* = 3 independent experiments). Confocal micrographs of Schwann cells cultured on (**e**) PCLm film and (**f**) TCP controls labelled for Syto-9 (green) and propidium iodide (red) for live/dead analysis (scale bar = 100 µm). The images were quantified to determine the cell viability as a percentage of live versus dead cells (**g**) (mean ± SD, *n* = 3 independent experiments).

As rat primary Schwann cells play an important role in peripheral nerve regeneration after injury, the Schwann cell phenotype and cell viability were assessed on the PCLm material (Figure 13a). Schwann cells cultured on PCLm films had an average length of 78 ± 10 µm (Figure 13c) and an aspect ratio of 4.5 ± 2.3 (Figure 13d). No statistical differences were detected compared to the cells grown on a tissue culture poly-styrene (TCP) control. The live/dead analysis was developed, followed by measuring the average Schwann cell length by confocal micrographs (mean ± SD, *n* = 3 independent experiments) of rat primary Schwann cells cultured on PCLm spin coated films (Figure 13e–g). The cell viability was determined as a percentage, 92 ± 6% (Figure 13g). This shows that PCLm was able to support Schwann cell proliferation and maintain a mature Schwann cell phenotype. 

### 3.5. In Vivo Model for a 6 mm Gap in a Rat Sciatic Model

As a preliminary test to study how the implant behaved in vivo, eight 50:50 PCLm:NaCl porogen conduits were fixed to the injured nerve through a silicon plug and filled with fibrin/thrombin (As an alternative to the use of fibrin, direct suturing was attempted but proved not to be suitable due to the breakage of microchannels). 

**Figure 14:**
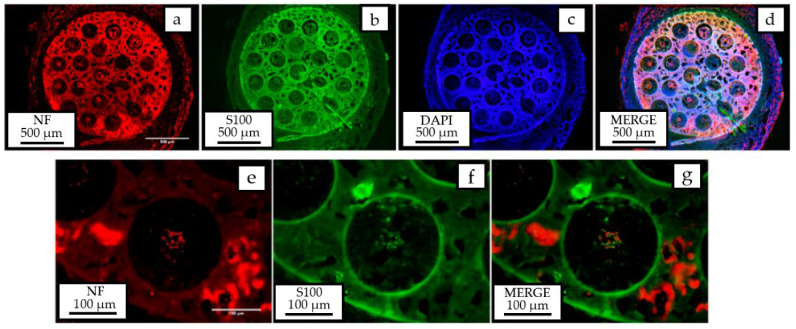
Immunohistochemical analysis showing the presence of myelinated axons (neurofilament 200-red), Schwann cells (S100*β*-green), and cell nuclei (DAPI-blue) observed in the inner part of microchannels.

**Figure 15:**
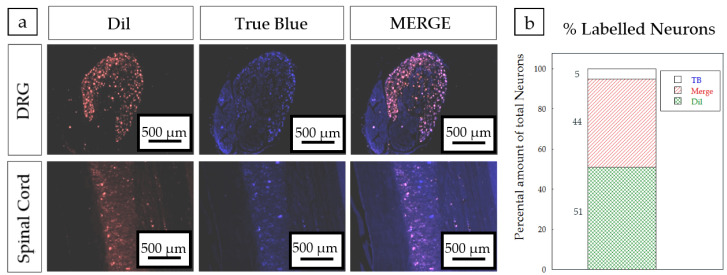
(**a**) Back labelled sensory neurons in the DRG (upper panels) and motoneurons (lower panels) in red (DiI), blue (True Blue) and merged. Scale bar = 500 µm. (**b**) Schematic representation of the tracer distribution in the DRG of implanted PCLm prototypes.

After 4 weeks, the regenerated nerves were collected and the mid-part of the tube was analyzed by immunohistochemistry for neurofilament 200, S100*β,* and DAPI fluorescent markers. Figure 14 clearly shows the presence of myelinated axons and Schwann cells, responsible for guiding axon growth and myelinate inside the microchannels. Axons can also be observed outside, possibly due to the separation between the proximal stump and the entrance of the PCLm implant. A high percentage of co-labelling (44%) was found between the nerve tracers used in the tibial and peroneal branches of the sciatic nerve (Figure 15), which is suggestive of a possible mismatch between the sprouting axons created by the space between the sutured nerve and the implant. 

## 4. Conclusions

Polycaprolactone was successfully methacrylated to obtain photocurable methacrylated polycaprolactone (PCLm). PCLm was employed for the microfabrication of implantable nerve guidance conduits based on a UV-casting process. This process was optimized to fabricate up to 50 mm long implants comprised of pass through microchannels. Microchannels were uniformly distributed through the implant section and could be as narrow as 200 µm in diameter. The fabrication protocol was compatible with the introduction of porosity using conventional leachable porogenerators, such as sodium chloride or glucose, where the weight percentage could be adjusted to vary the properties of the implant, pursuing the objective of mimicking the native mechanical properties of the nerve. Moreover, by means of high internal phase emulsion (HIPE), implants with highly porous sponge-like structures were also obtained. The 3D conduit made of PCLm showed a good biocompatibility and a suitable biodegradation rate. The biomaterial also showed good neuroregeneration properties for neuronal cell proliferation and differentiation. Finally, the implant was validated in a short gap (6 mm) rat sciatic model, showing the presence of myelinated axons and Schwann cells in the lumen of the microchannels.

Further studies, such as histocompatibility and hemolysis tests, should be also developed to accordingly evaluate either the presence of non-observed inflammatory effects during the histological analysis or unlikely induced damage on red blood cells. Future assays in nerve guide implants will continue with the combination of the developed multichannel array structure surrounded by a hollow porous tube 1 mm longer in the edges, to allow the coaptation of the nerve stumps close to the inner microchannels. The addition of a hollow tube to the implant should provide good mechanical behaviour to function as protection for the inner microchanneled conduit. Research should also combine the biocompatibility with a biodegradation rate in the range of the figures reported in this work. This combined approach could pave the way toward the application for regeneration of long gap injuries in humans.

## Supplementary Materials

The following are available online at https://www.mdpi.com/2073-4360/12/4/971/s1, Figure S1: NMR spectrum of PCL and PCLm, Figure S2: SEM image of microchanneled PCLm tube showing the 200 µm diameter channels.
