# Supplementary material for: UV-Casting on Methacrylated PCL for the Production of a Peripheral Nerve Implant Containing an Array of Porous Aligned Microchannels
Source: Polymers (Basel). 2020 Apr 22;12(4):971. doi: 10.3390/polym12040971 (PMC7240584; doi:10.3390/polym12040971)
Supplement: Supplementary file 1 [file polymers-12-00971-s001.zip › polymers-749655-supplementary.pdf]

polymers

Supplementary material:

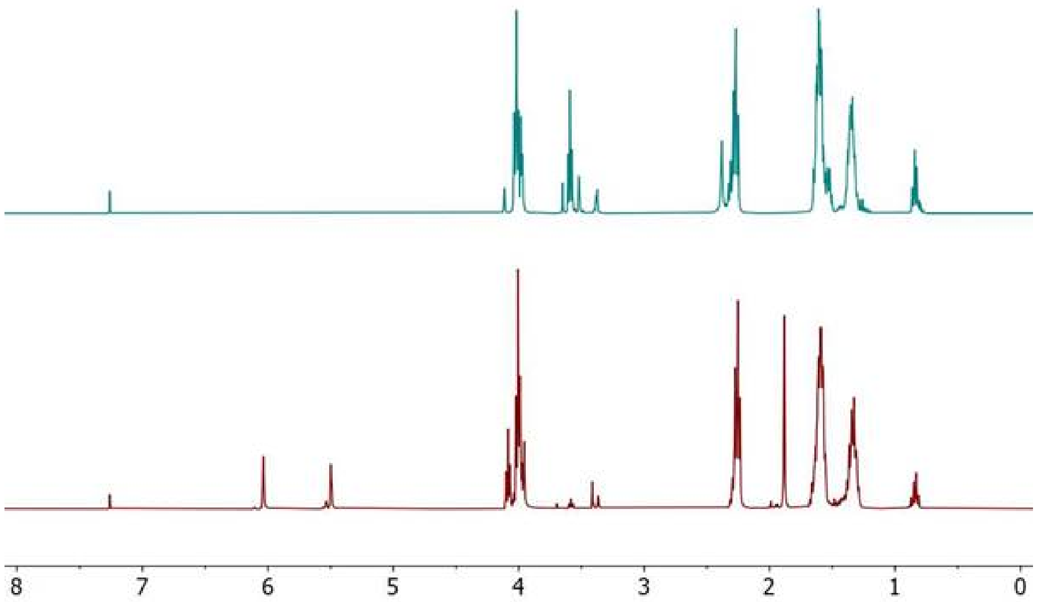

Figure S1 NMR spectrum of PCL (top) and PCLm (bottom). All peaks are referenced to the diluent $CDCl_3$

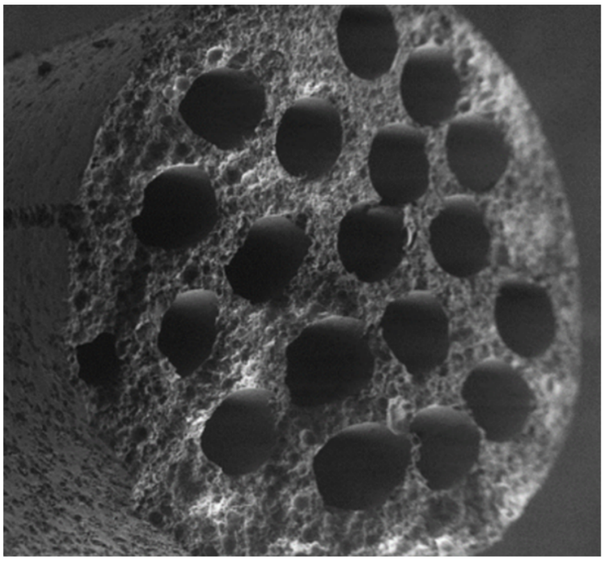

Figure S2 SEM image of microchanneled PCLm porous tube showing the 200 µm diameter channels and their arrangement over the tube section.

© 2019 by the authors. Submitted for possible open access publication under the terms and conditions of the Creative Commons Attribution (CC BY) license (http://creativecommons.org/licenses/by/4.0/).
